# Radiosurgery for Patients with More Than Ten Brain Metastases

**DOI:** 10.7759/cureus.6728

**Published:** 2020-01-21

**Authors:** Yoshihisa Kida, Yoshimasa Mori

**Affiliations:** 1 Neurological Surgery, Ookuma Hospital, Nagoya, JPN; 2 Radiation Oncology and Neurosurgery, Shin-Yurigaoka General Hospital, Kawasaki, JPN

**Keywords:** gamma knife, multiple, srs, srt

## Abstract

Purpose

In this article, we report on Gamma Knife radiosurgery performed on patients with more than 10 brain metastases. Although the standard treatment for multiple brain metastases is currently believed to be whole-brain irradiation, many patients are averse to it due to the potential for serious complications such as cognitive impairment.

Cases and Methods

Here, 70 cases of Gamma Knife radiosurgery for metastatic foci originating from various primary cancer are reviewed. Several different treatment protocols were selected: (1) single session, (2) two or three consecutive sessions, (3) fractionated irradiation for large tumor and stereotactic radiosurgery (SRS) for small ones, and (4) salvage treatment for recurrent tumors after whole-brain irradiation.

Results

Despite the long beam-on-time (BOT) necessary for Gamma Knife radiosurgery and unavoidable spillage irradiation to the entire brain, all the treatments were completed without any major difficulties.

Conclusion

SRS or radiotherapy might be a treatment choice for patients with more than 10 brain metastases. However, the very long treatment time and big spillage irradiation to the entire brain warrants that large metastatic foci should be removed before or after radiosurgery.

## Introduction

The current treatment choice for multiple brain metastases is whole-brain irradiation. With the aid of stereotactic radiosurgery (SRS), current studies have demonstrated that the presence of fewer than five brain metastasis is associated with excellent tumor control. Moreover, a few reports have concluded that radiosurgery for fewer than 10 brain metastases is not inferior to radiosurgery for fewer than five brain metastases in terms of tumor control [1,2]. However, the treatment of more than 10 brain metastases seems to be more difficult and challenging. For example, the number of metastatic foci may increase considerably during the waiting period prior to the scheduled radiosurgery. Both SRS and stereotactic radiotherapy (SRT) are often required because of differences in tumor size and location. In other cases, tumors may recur after whole-brain radiation therapy (WBRT), or patients may strongly refuse WBRT. Accordingly, appropriate responses to these various situations and complications are needed. Here, we report and compare the treatment of more than 10 brain metastases using several treatment methods and different treatment intervals. Finally, we would like to discuss how SRT or SRS can contribute to managing such difficult situations in combination with other treatment modalities.

## Materials and methods

Since the installation of a Gamma Knife device at our institute (Ookuma Hospital, Nagoya, Japan) in May 2016, we have treated 70 individuals with more than 10 brain metastases each; our findings are reported in the present study. Among these patients, some had experienced recurrent brain metastases after WBRT, some had refused to undergo WBRT, and the others had a few large brain metastases accompanied by multiple smaller metastatic foci. Many of these patients were treated with a single session of Gamma Knife radiotherapy or with two or three consecutive sessions of staged stereotactic irradiation at time intervals of less than two weeks (Table 1). 

**Table 1:** Details of Irradiation methods used in this study SRS: stereotactic radiosurgery

Irradiation methods used in this study
1. Multiple small metastases treated in a single session
2. Multiple small or numerous tumors treated consecutively within two or three days
3. Multiple tumors including several or a few larger ones with a mean diameter of more than 30 mm are treated using staged radiotherapy with a two-week interval. Meanwhile, the smaller tumors are treated using SRS
4. Even after whole-brain irradiation, metastatic tumors may often reappear. In this situation, only growing tumors are treated using SRS

There were 31 males and 39 females, ranging in age from 24 to 89 years with a mean age of 64.8 years. Many of metastases were from lung cancer (50), followed by breast cancer (9), and others (11). Some patients underwent WBRT either before or after the stereotactic procedure. In such situations, the dose accumulation from the additional SRS needed to be taken into account. The follow-up periods ranged from 1 to 32 months, with a mean of 7.7 months. The tumor pathology, indications, and other characteristics of the groups are shown below (Table 2).

**Table 2:** Characteristics of the patients involved in this study

No	Characteristic	Details
1	Gender	Male:31; female: 39
2	Age	24–89 years (mean: 64.8 years)
3	Pathology	Lung: 50 (Adeno: 36; small: 7; non-small: 2; unknown: 5)
		Breast: 9
		Colon: 1
		Renal: 2
		Uterine or ovary: 2
		Others: 6
4	Follow-up	1–32 months (mean: 7.7 months)

Follow-up studies were performed in terms of tumor control as well as the number of newly appearing metastases. Careful check-up data for radiation injury were recorded and compared. In this study, the results of each of the four groups were compared. The number of tumor treatment and the time duration were recorded. The beam-on-time (BOT) refers to the time during which the patients must remain under the Gamma knife device. The dose rate of the Gamma Knife ranged from 3.3 to 2.3 Gy per minute at our institute during the study period.

All statistical analyses of data were performed using the statistical software package JMP 9.02 (SAS Institute, Cary, NC). Statistical differences between the four groups were studied with the Student's t-test. A p-value of <0.05 was considered statistically significant. Overall survival was not studied because the follow-up periods were short. Our main objective was to explore how we could manage multiple metastases of more than 10, and how the patients reacted to the procedures. 

## Results

**Figure 1:**
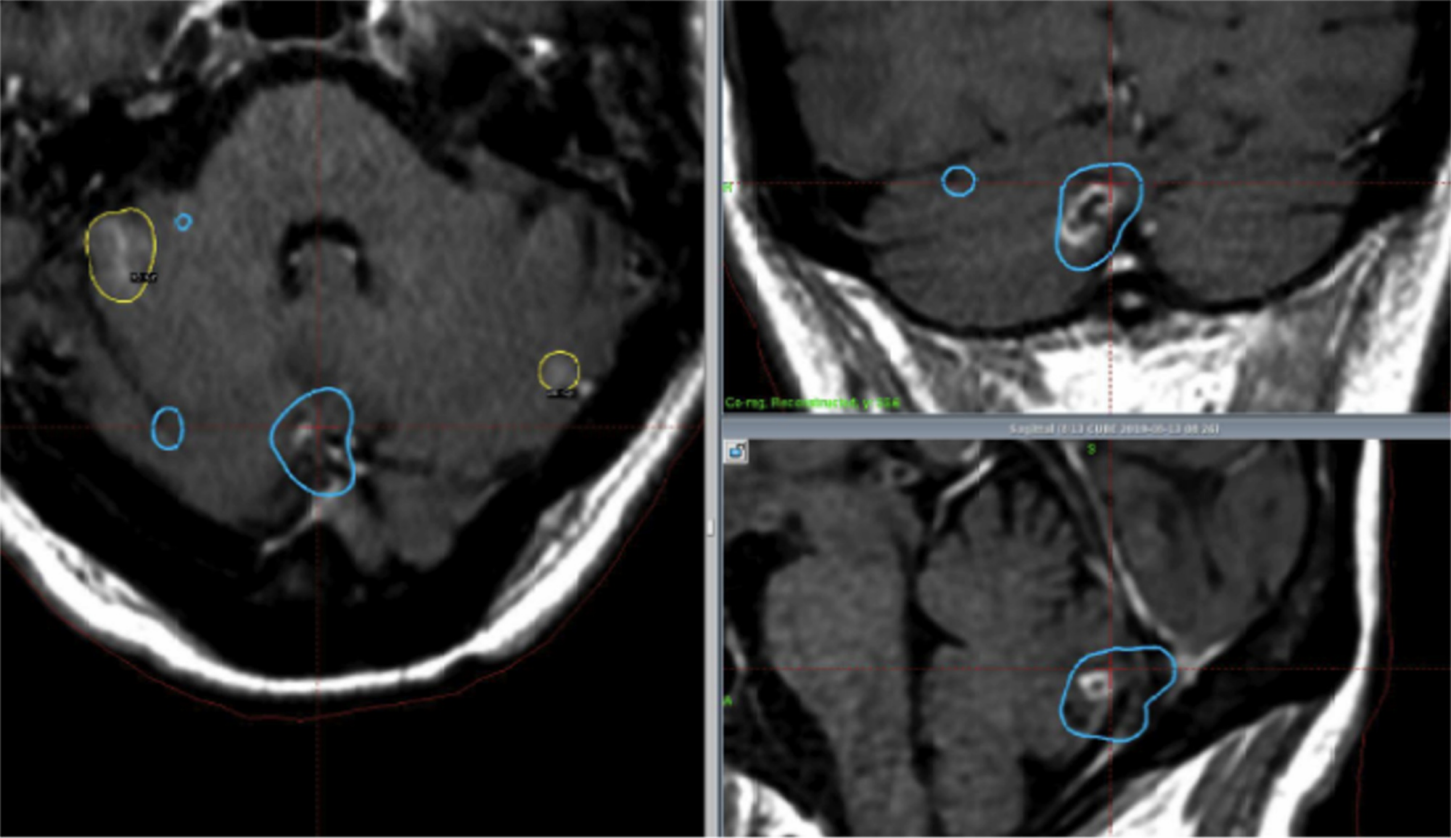
Images of the treatment methods for group A Blue markings indicate the tumor sites in the first treatment; yellow markings indicate the tumors treated in the second treatment

**Figure 2:**
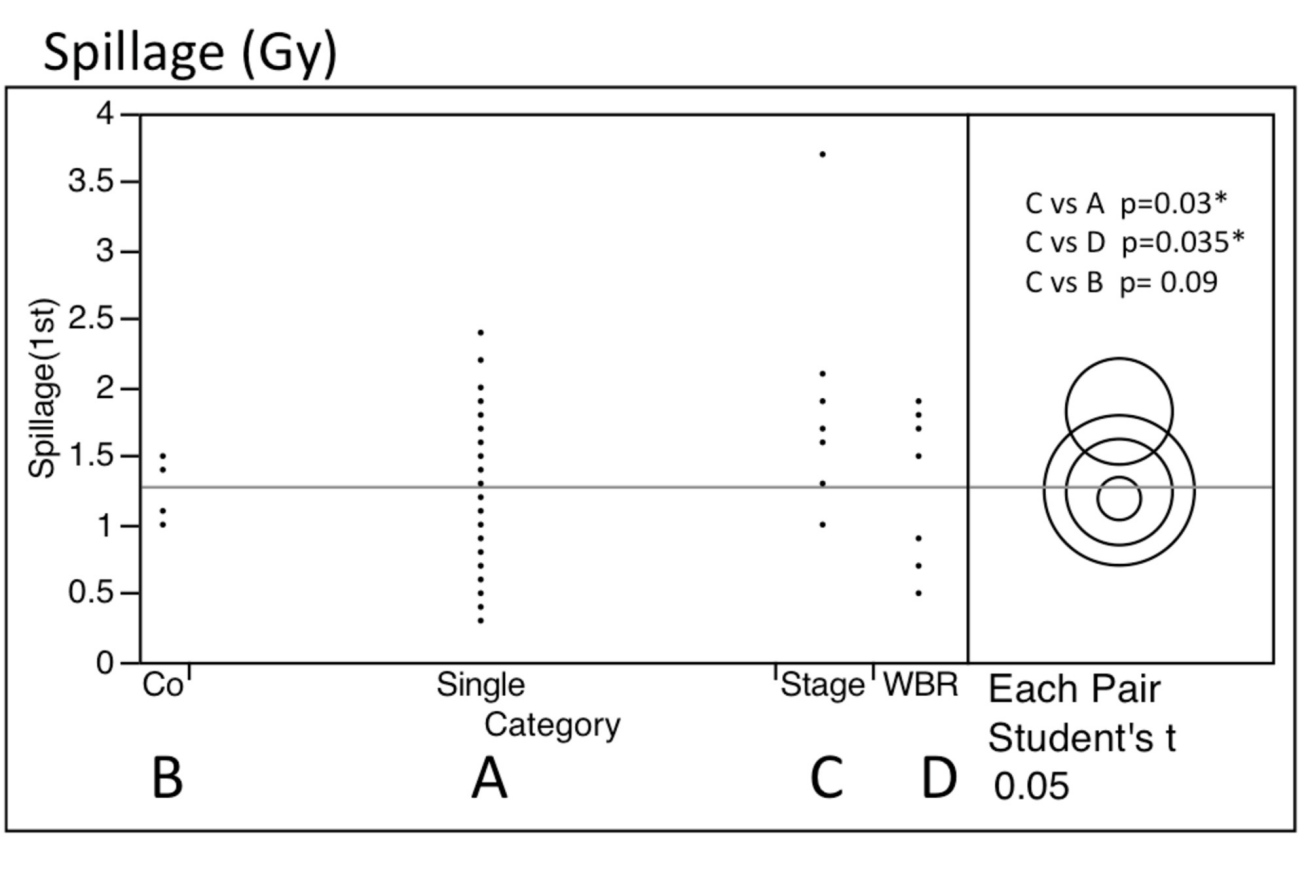
Distribution of spillage for the four treatment groups

For small tumors, marginal doses between 16-20 Gy were selected and delivered. Marginal doses between 10-12 Gy were used for two-staged treatments, which was equivalent of 15-17 Gy delivered in a single session. In general, the tumor control for tiny metastatic foci was sufficient and further treatment of irradiated foci was not required (Figure 1). However, new metastatic foci, requiring further treatment were often encountered. In groups A, B and C, WBRT or additional SRS were required in some cases. In general, the number of tumors was smaller for second treatment procedures. BOT ranged from 160 to 185 minutes for the first SRS, decreasing to less than 150 minutes for the second, and 147 minutes for the third. This was also the case in the second treatment, except for group C with staged treatment (Figure 2).

**Figure 3:**
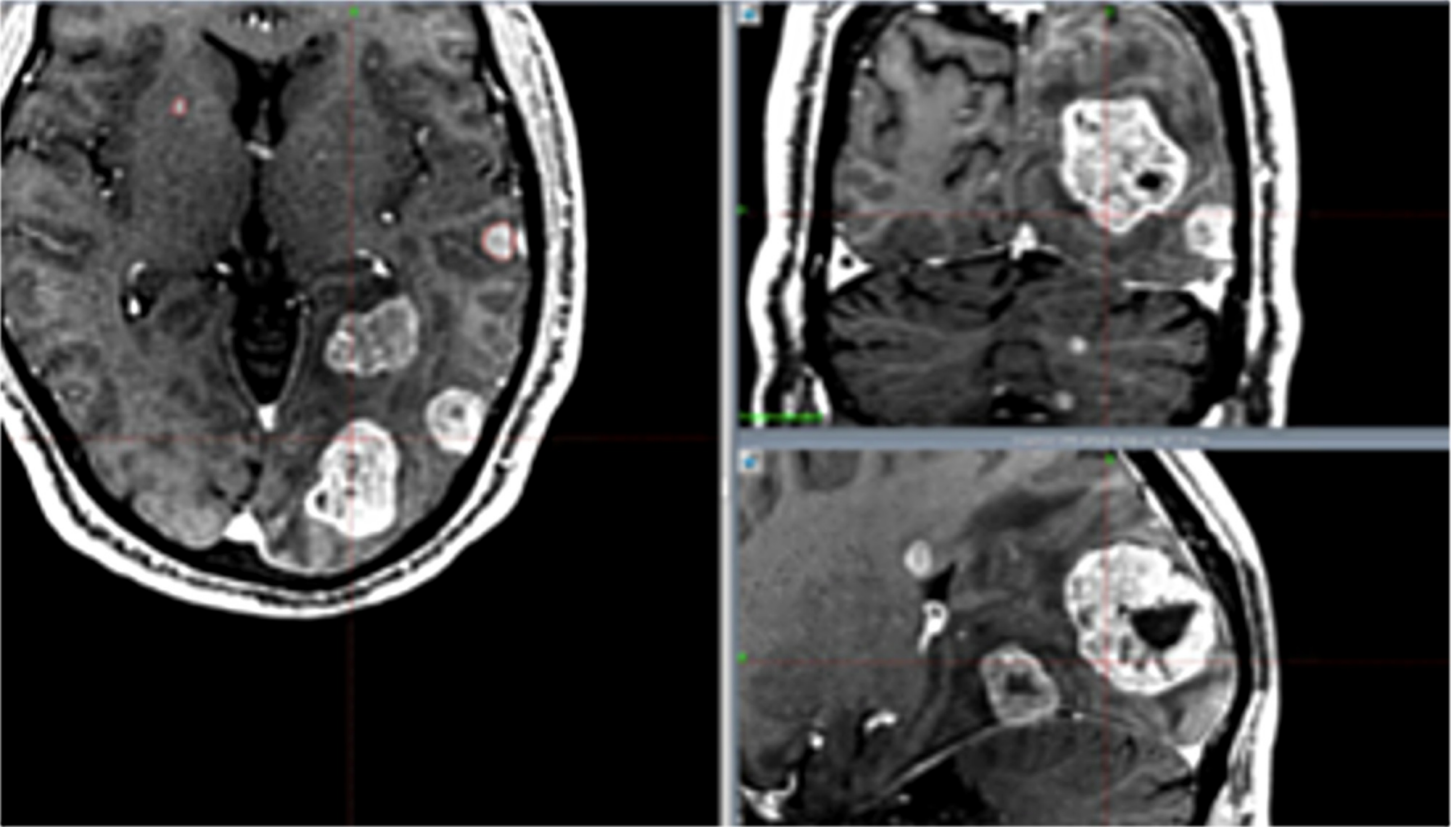
Images of group C treated with staged treatment (first radiosurgery)

Conversely, the mean dose for the entire brain spillage was low in groups A and D. Despite the long radiation time, patients were able to tolerate the entire treatment procedure. The BOT and spillage dose during the first treatment is shown below (Figure 3). The mean spillage was significantly larger in group C with staged treatment than the other groups (p: <0.05).

The longest BOT (minutes), the largest spillage (Gy), and the largest amount of energy (Joule) were recorded in group C in the staged treatment with large tumors (Table 3).

**Table 3:** Number of tumors, beam-on-time, spillage, and energy in each treatment group SRS: stereotactic radiosurgery; BOT: beam on time; WBRT: whole-brain radiation therapy

Group	A) Single session	B) Consecutive	C) Staged plus single	D) After WBRT
Cases	50	4	8	8
Age, years	64.8	56.5	65.7	67.4
Tumors at first SRS (mean)	18.3	23.8	11.6	15.6
BOT at first SRS, minutes	176.9	184.5	163.0	171.1
Spillage at first SRS, Gy	1.18	1.25	1.90	1.23
Energy at first SRS (Skull), J	4.0	4.7	6.3	4.4
Tumors at second SRS (mean)	15.7	14.5	13.3	–
BOT at second SRS, minutes	143.5	114.7	146.1	–
Spillage at second SRS, Gy	1.00	0.83	1.66	–
Tumors at third SRS (mean)	17.0	6	26.7	–
BOT at third SRS, minutes	142.3	104.2	188.7	–
Spillage at third SRS, Gy	1.37	0.65	1.37	--
Total spillage, Gy	3.56	2.73	4.94	1.93 + 30 (WBRT, 10 Fractions)
Outcome and comorbidity	Some required SRS more than four times	Some required WBRT after RS	Lot of neurological death recorded along with longest BOT and largest spillage	Cognitive impairment occurred in several cases

**Figure 4:**
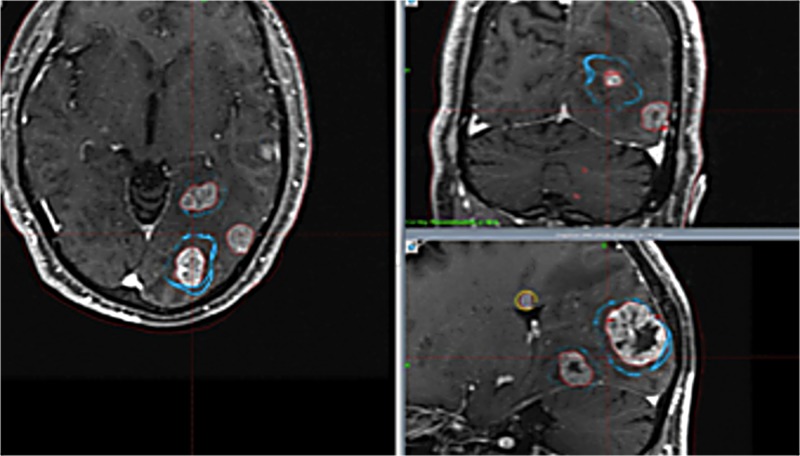
Images of group C treated with staged treatment (second radiosurgery) Blue line shows the tumor margin at the first stage. The red line shows the tumor margin at the second treatment. The yellow line indicates the tumor margin treated with single radiosurgery. Large tumors became much smaller after the first stage of treatment

**Figure 5:**
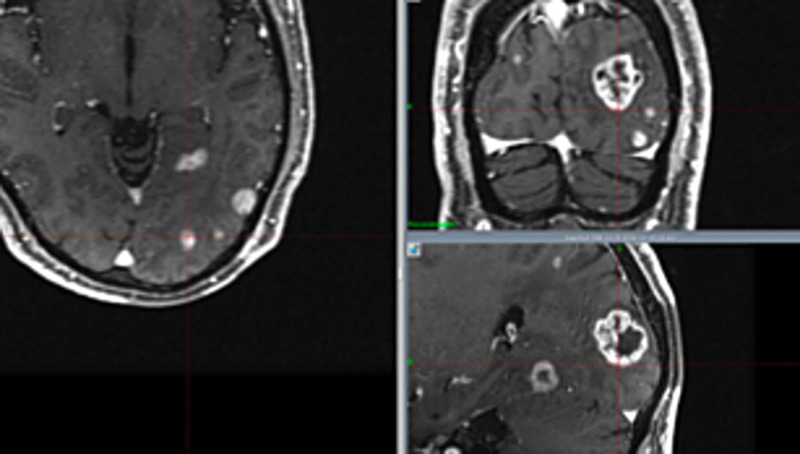
Images obtained after one month of second treatment for group C

The spillage and energy in group C were significantly larger than those in groups A and D (p: <0.05). The follow-up periods have not yet been long enough, and the data of overall survival are not available. However, so far no serious adverse effects have been encountered except for cognitive impairment in group D. Tumor control was insufficient at times for large tumors, resulting in many neurological deaths in group C. At the first treatment in group C, several large tumors were treated with a staged method, with two weeks' interval between stages. Meanwhile, several small tumors were treated with SRS (Figure 3). Tumors showed remarkable shrinkage after the first SRS. This time, several small tumors were treated with SRS again (Figure 4). Excellent tumor control was obtained after one month, but a subsequent neurological deterioration occurred after several months (Figure 5).

 

Subsequent WBRT was required in several cases. The recurrent tumors that occurred after WBRT showed excellent responses after additional radiosurgery.

## Discussion

At present, SRS and SRT are considered key treatments for metastatic brain tumors [1,2,3]. Several patients with fewer than 5 or 10 brain metastases have been successfully treated using radiosurgery [2]. Currently, WBRT is selected for cases with more than 10 or numerous metastases [4,5]. However, it is often difficult to perform WBRT because of the risk of cognitive impairment or the poor general condition of some patients. In fact, many patients dislike or refuse WBRT. SRS or staged treatments have been used for various different situations in terms of multiplicity, location, and condition of primary cancer as well as tumor dissemination or metastatic tumors in the other organs. Cases with more than 10 brain metastases face many more difficulties than those with fewer than 10 metastases. Numerous shower metastases or dissemination may occur in the clinical course. Because of tumor multiplicity, the time required for the treatment may be long enough with stereotactic radiation. The total dose of spillage, or energy leakage of the dose to the entire brain, is among the serious matters not to be neglected. 

In this paper, we reviewed a few strategies for treating individuals with more than 10 brain metastases. For the treatment of multiple small metastatic foci, either single or consecutive sessions can be selected. Long treatment time is required to complete consecutive treatments, but tumor control is often attained. Further treatments for newly appearing metastatic foci are often necessary. When one or a few large metastases are accompanied by multiple small tumors, staged treatment with an interval of two weeks seems adequate, but it takes a very long time to complete the treatment in association with significantly increased spillage. Conversely, for recurrent tumors after WBRT, only growing tumors were treated in our series. In our experiences, however, it was not difficult to control recurrent tumors without causing late complications in the surrounding brain tissue. When large brain tumors are involved and managed with staged treatment, a large amount of spillage and energy may happen; such situations often warrant longer treatment time as well. Hence, the surgical resection of large tumors before or after radiosurgery can be considered.

Several investigators have mentioned that treatment with SRS alone or with SRS + WBRT in patients with newly diagnosed brain metastases results in similar overall survival outcomes. Therefore, the addition of WBRT did not always improve the overall survival or reduce the average total treatment cost. This indicates that radiosurgery might be useful not only in terms of tumor control but also in terms of cost-effectiveness [6]. Some studies have postulated treatment strategies using hypo-fraction or staged methods as well [7,8].

## Conclusions

For the treatment of more than 10 brain metastases, various treatment strategies have been considered and proposed. In addition to single-session treatments, a consecutive or staged strategy can also be chosen. Among the recurrent tumors after WBRT, only growing ones should be treated with SRS. Although a long treatment time for recurrent tumors and increased radiation spillage to the entire brain have been recorded, patients with more than 10 brain metastases could tolerate the procedures without any serious complications. Instead of WBRT, multiple brain metastases could be treated with both SRT and SRS. When a few large tumors are involved, surgical resection has to be considered before or after radiosurgery.

## Appendices

The authors’ contributions to the study and manuscript preparation include the following:

Study conception and design, and acquisition of data - Y Kida.

Analysis and interpretation of data - all authors.
